# Fibroblast morphology, growth rate and gene expression in facial melasma^☆^

**DOI:** 10.1016/j.abd.2021.09.012

**Published:** 2022-07-12

**Authors:** Ana Cláudia Cavalcante Espósito, Gabrielli Brianezi, Luciane Donida Bartoli Miot, Hélio Amante Miot

**Affiliations:** a Department of Dermatology and Radiotherapy, Faculty of Medicine, Universidade Estadual Paulista, Botucatu, SP, Brazil; b Department of Pathology, Faculty of Medicine, Universidade Estadual Paulista, Botucatu, SP, Brazil

**Keywords:** Aging, Collagen, Melasma, Pigmentation disorders, Ultraviolet rays

## Abstract

**Background:**

In addition to melanocytic hyperfunction, changes are observed in the upper dermis of melasma, and fibroblasts play a central role in collagen synthesis and pigmentation induction.

**Objective:**

To explore the morphology, growth rate, and gene expression profile of fibroblasts from the skin with melasma in comparison to fibroblasts from the adjacent healthy skin.

**Methods:**

Ten women with facial melasma were biopsied (lesion and adjacent healthy skin), and the fragments were processed for fibroblast culture. Samples from five participants were seeded to evaluate growth (days 2, 5 and 8) and senescence (SA-β-gal) curves. The samples from the other participants were submitted to real-time PCR to comparatively evaluation of the expression of 39 genes.

**Results:**

Cultured fibroblasts from melasma skin were morphologically less fusiform in appearance and on average a 34% (95% CI 4%‒63%) greater proportion of cells labeled with SA-β-gal than the fibroblasts from the adjacent skin. The cell growth rate was lower for the melasma samples after eight days (p < 0.01). The*WNT3A, EDN3, ESR2, PTG2, MMP1,* and *SOD2* genes were up-regulated, whereas the *COL4A1, CSF2, DKK3, COL7A1, TIMP4, CCL2,* and *CDH11* genes were down-regulated in melasma skin fibroblasts when compared to the ones from adjacent healthy skin.

**Study limitations:**

Small sample size; absence of functional tests.

**Conclusions:**

Fibroblasts from the skin with melasma showed a lower growth rate, less fusiform morphology and greater accumulation of SA-β-gal than those from adjacent photo exposed skin. Moreover, their gene expression profile comprised factors that may contribute to upper dermis damage and sustained melanogenesis.

## Introduction

Melasma is a highly prevalent acquired dyschromia, which results from an increase in the activity of the epidermal melanin unit.^1^ Lesions affect photo exposed areas (e.g., the face), especially in women during menacme. Its pathogenesis is not yet fully understood, and the role of basement membrane zone alterations, damage to the upper dermis, and fibroblast activity in the development of the disease are posed as recent research questions.^2^, ^3^, ^4^, ^5^

Compared with the adjacent healthy skin (photoexposed), the skin with melasma has an increased amount of epidermal melanin, more mature melanosomes, hypertrophied melanocytes, and prominent solar elastosis. There is also an increase in the number of vessels and mast cells, as well as increased expression of inflammatory mediators, such as iNOS, NF-Kβ, endothelin, and growth factors, such as VEGF, and growth factors derived from hepatocytes,-stem cells, fibroblasts and nerves.^6^ Pendulum melanocytes – basement layer melanocytes that project toward the upper dermis – and basement membrane alterations have also been described in melasma.^2^ There is a decrease in type I collagen associated with an increase in metalloproteinase activity (MMP 1, 2, 3, and 9).^7^ Some genes related to lipid metabolism are down-regulated in melasma skin and the skin barrier function is impaired.^8^

Sun exposure is the main risk factor for the development of melasma. Chronic photoexposure, in addition to melanogenesis, induces oxidative stress, which promotes cellular senescence.^5^ In addition to photoaging and intrinsic aging, melasma has a more prominent senescent phenotype in affected skin. Ultraviolet radiation (UVR)-induced early cell senescence promotes functional alterations that can trigger and perpetuate melanogenesis.^9^, ^10^

Fibroblasts play a central role in pigmentation and their transition to a senescent profile promotes a modification of their autocrine and paracrine activity.^10^ In melasma skin, fibroblasts release more stem cell-derived factors and its epidermal receptor c-kit.^11^ Senescent fibroblasts can express inflammatory and melanogenic factors, which can lead to the development of melasma.^3^

The senescent phenotype was identified in fibroblasts with melasma more intensely than in adjacent photo exposed skin, based on the immunoexpression of p16^INK4A^, which may justify the stimulus for sustained skin pigmentation.^9^, ^10^ However, to date, the potential for cell replication has not been explored, and the gene expression profile of these dermal fibroblasts has not been characterized regarding the growth factors and tissue repair, activation of the WNT/β-catenin (WNT) tissue growth pathway, neocollagenesis, metalloproteinase synthesis, estrogen receptors, and antioxidant mechanisms. This characterization can support pathophysiological models and treatment strategies.

This study aimed to explore the morphology, growth rate and gene expression profile of fibroblasts from facial melasma, compared to adjacent photoexposed skin.

## Methods

This project was approved by the Research Ethics Council (Unesp, Botucatu-SP, number 0461-11). and all participants consented to participate.

Ten adult women with facial melasma underwent biopsy (2-mm punch, using a sterile technique) of the melasma skin on the malar region and from the adjacent, clinically normal, photoexposed skin (<2 cm of distance), laterally. The participants were untreated for the dermatosis for at least 30 days, except for the use of sunscreen.

The skin fragments were sectioned and placed in fibroblast culture, maintained in medium 106 (Gibco™) containing growth factors (LSGS Kit, Gibco™). The fibroblasts were seeded (in triplicate) at a density of 5 × 10^3^ in a 12-well cell culture plate.

Cultured fibroblasts, from healthy and damaged skin, from five participants, were seeded at a density of 1 × 10^4^ in a 6-well cell culture plate, and the number of cells per cm^2^ was evaluated after two, five and eight days, based on the evaluation of 30 fields, and compared (between topographies) by a generalized linear mixed-effects model. Sample normality was evaluated by the Shapiro-Wilk test.

Subsequently, cell senescence was evaluated using senescence β-galactosidase (SA-β-gal) staining kit (Cell Signaling Technology®), following the manufacturer's instructions. After 24 hours of cell plating, the culture medium was removed, followed by washing with PBS and the addition of fixation solution for 15 minutes at room temperature. Then, it was washed with PBS twice, followed by overnight incubation with β-galactosidase solution (Cell Signaling Technology®) at 37 °C. After the solution was removed, 70% glycerin was added.

A total of 300 cells were counted per sample. The morphology of the cultured fibroblasts and the percentage of cells labeled with cytoplasmic SA-β-gal were compared between the topographies (melasma and adjacent healthy skin).

Fibroblasts from the primary cell culture of the other five participants were submitted to a real-time PCR array (96-well plate, Custom RT^2^ Profiler PCR Arrays, Qiagen) to assess the expression of 39 genes associated with growth factors and tissue repair, WNT pathway activation, neocollagenesis, metalloproteinase synthesis, estrogen receptors, and antioxidant mechanisms (Table 1). Total RNA was obtained with the RNase Mini Kit (Qiagen), and the RNA reverse transcription was performed using RT^2^ First Strand Kit for RT-PCR (Qiagen), following the manufacturer's instructions.

**Table 1:** List of the 39 genes assessed in the study, and their main function in the skin.

Gene	Name	Main function (skin)
*CCL2*	C-C Motif Chemokine Ligand 2	Chemokine involved in the tissue repair process
*CDH11*	Cadherin 11	Related to cell adhesion and epithelial repair
*CDKN2A*	Cyclin-dependent kinase inhibitor 2A	Cell response to inflammatory and neoplastic stimulus
*COL4A1*	Type IV collagen	Basement membrane component
*COL7A1*	Type VII collagen	Anchoring fibril component
*CSF2*	Colony-stimulating factor 2	Involved in the process of epithelial repair
*DKK1*	Dickkopf-related protein 1	Wnt/β-catenin cell growth pathway inhibitor
*DKK3*	Dickkopf-related protein 3	Wnt/β-catenin cell growth pathway inhibitor
*EDN1*	Type 1 endothelin	Induces melanogenesis and vascular proliferation
*EDN3*	Type 3 endothelin	Induces melanogenesis and vascular proliferation
*ESR1*	Estrogen Receptor 1 (α)	Estrogen receptor linked to the canonical pathway
*ESR2*	Estrogen Receptor 2 (β)	Estrogen receptor linked to tissue repair
*FGF2*	Fibroblast growth factor type 2	Tissue damage repair, mitotic for fibroblasts
*GLB1*	Beta-galactosidase 1	Constitutional gene. Experiment control
*HGF*	Hepatic growth factor	Tissue damage repair
*IL1A*	Interleukin 1a	Primary inflammatory skin response
*IL1B*	Interleukin 1b	Primary inflammatory skin response
*IL6*	Interleukin 6	Primary inflammatory skin response
*MAPK14*	Mitogen-activated protein kinase 14	Cell response to inflammatory stimulus
*MIF*	Macrophage migration inhibitory factor	Primary inflammatory skin response
*MMP1*	Matrix metalloproteinase type 1	Degradation of type I, II and III collagen
*MMP2*	Matrix metalloproteinase type 2	Degradation of type IV collagen
*MMP7*	Matrix metalloproteinase type 7	Extracellular matrix degradation
*MMP9*	Matrix metalloproteinase type 9	Extracellular matrix degradation and angiogenesis
*NGR1*	Type 1 neuregulin	Regulates melanocytic growth and skin color
*OXR1*	Oxidative resistance protein type 1	Cell response to oxidative stress
*OXSR1*	Oxidative stress protein type 1	Cell response to oxidative stress
*PTGS2*	Cyclooxygenase type 2	Prostaglandin E2 synthesis
*SOD1*	Superoxide dismutase type 1	Protects the cell from active oxygen species
*SOD2*	Superoxide dismutase type 2	Induced in response to mitochondrial oxidative stress
*TIMP1*	Tissue inhibitor of metalloproteinases 1	Inhibitor of collagen I, II and III degradation
*TIMP2*	Tissue inhibitor of metalloproteinases 2	Inhibitor of collagen IV degradation
*TIMP3*	Tissue inhibitor of metalloproteinases 3	Inhibitor of collagen and extracellular matrix degradation
*TIMP4*	Tissue inhibitor of metalloproteinases 4	Inhibitor of extracellular matrix degradation
*TP53*	p53 protein	UVB photoaggression marker, anti-angiogenic
*VEGFA*	Vascular endothelial growth factor type A	Promoter of angiogenesis
*WIF1*	Wnt inhibitory factor-1	Inhibits the Wnt/β-catenin cell growth pathway
*WNT3A*	WNT family member 3A	Wnt/β-catenin canonical pathway activator
*WNT5A*	WNT family member 5A	Wnt non-canonical pathway activator

The effect size for gene expression (2^-ΔΔCt^) was estimated by the fold change (melasma/adjacent skin) for each participant.^12^ Fold changes were represented by their mean and 95%CI, estimated by 10,000 resamplings with accelerated bias correction (BCa).

## Results

The main clinical and demographic data of the assessed patients are shown in Table 2.

**Table 2:** Main characteristics of the ten patients with facial melasma submitted to skin biopsy on the region with malar melasma and adjacent photoexposed facial skin, whose samples were used for: growth curve, morphology and SA-β-gal evaluation, or gene expression test.

Growth curve, morphology and SA-β-gal evaluation					
Case	Age	Phototype	Time length of melasma (years)	Family history of melasma	mMASI
1	46	IV	10	Yes	9.5
2	46	IV	13	Yes	8.2
3	35	III	2	Yes	6.6
4	41	IV	11	No	11.5
5	38	IV	10	No	12.4

Gene expression test					
6	41	IV	23	No	9.9
7	38	IV	17	Yes	19.2
8	41	III	16	Yes	9.5
9	47	III	28	Yes	7.6
10	44	IV	5	No	10.1

mMASI (modified Melasma Area Severity Index.

**Figure 1:**
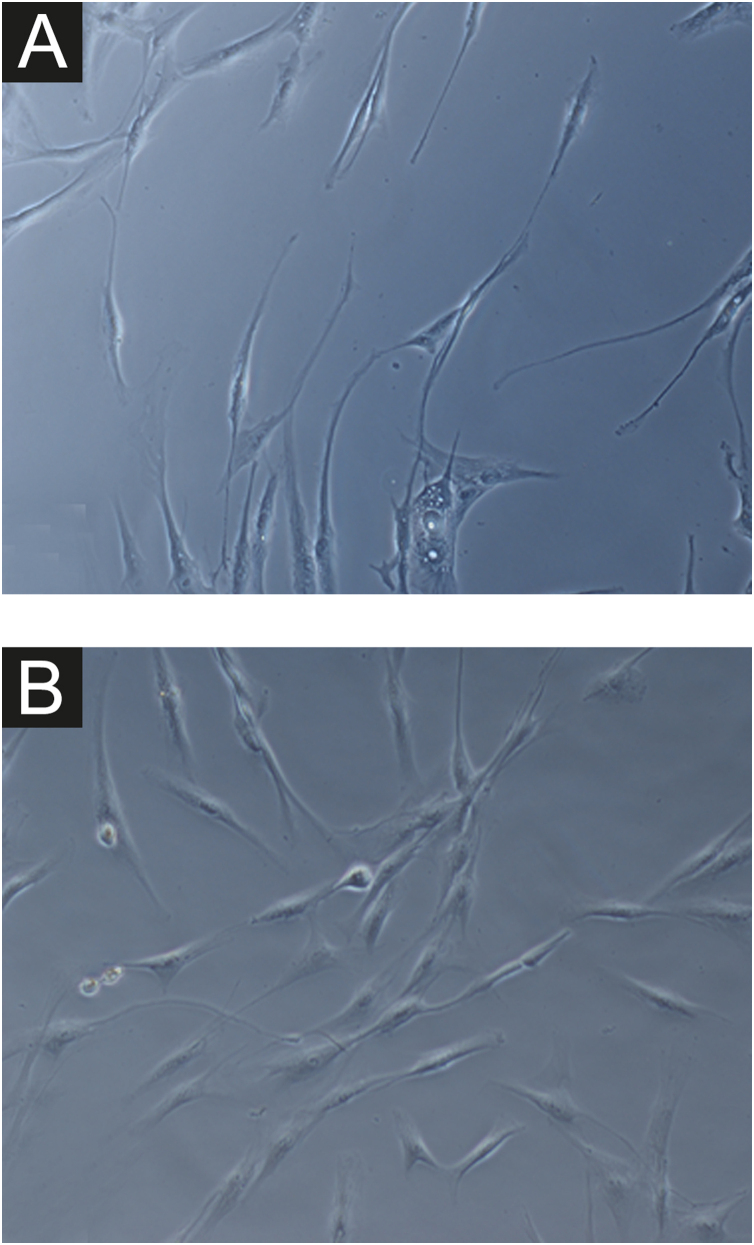
Phase-contrast microscopy showing fibroblasts from skin with facial melasma (A) and from adjacent healthy skin (B) in cell culture (SA-β-gal, ×400), showing lower cell density and less elongated (fusiform) wider morphology, in melasma.

**Figure 2:**
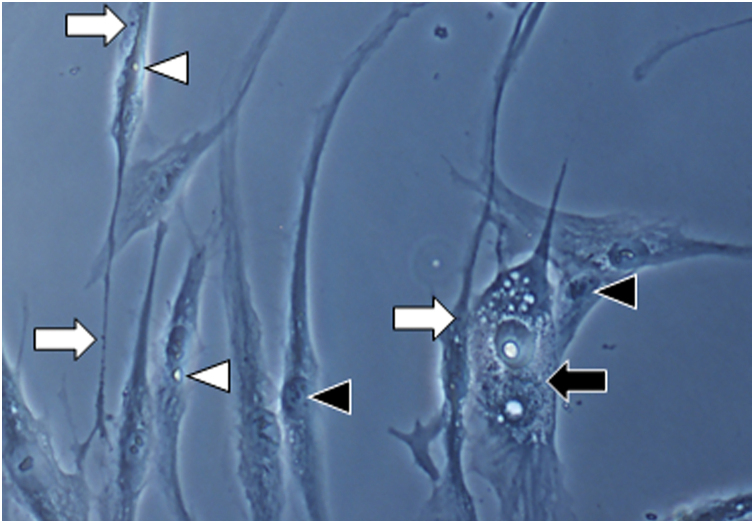
Fig. 1A magnification (×1000): senescent morphology of fibroblasts from skin with melasma, showing a higher proportion of juxtanuclear bodies (black arrow), frequent granular cytoplasmic structures (SA-β-gal+; white arrow), lipid droplets (white arrowhead), and segmented nucleoli (black arrowhead).

**Figure 3:**
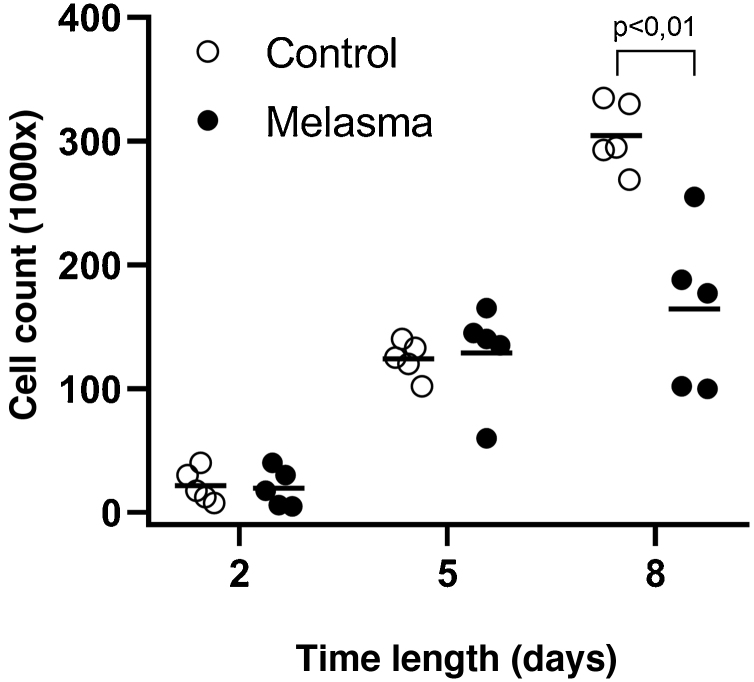
Fibroblast growth curves (cell count per cm^2^) for five patients: healthy skin and skin with facial melasma. Horizontal line bar represents the mean of each sample. The data are compared longitudinally by a generalized linear mixed-effects model.

In all samples, cultured fibroblasts from melasma skin showed lower cell density, and the fibroblasts were morphologically less elongated, wider, and less fusiform, in addition to showing more cells labeled with SA-β-gal (mean superiority of 34%; 95%CI 4%‒63%; p < 0.05) than cultures from adjacent skin (Figure 1, Figure 2). Moreover, the cell growth rate was lower for the melasma samples after eight days in culture (p < 0.01; Fig. 3).

**Figure 4:**
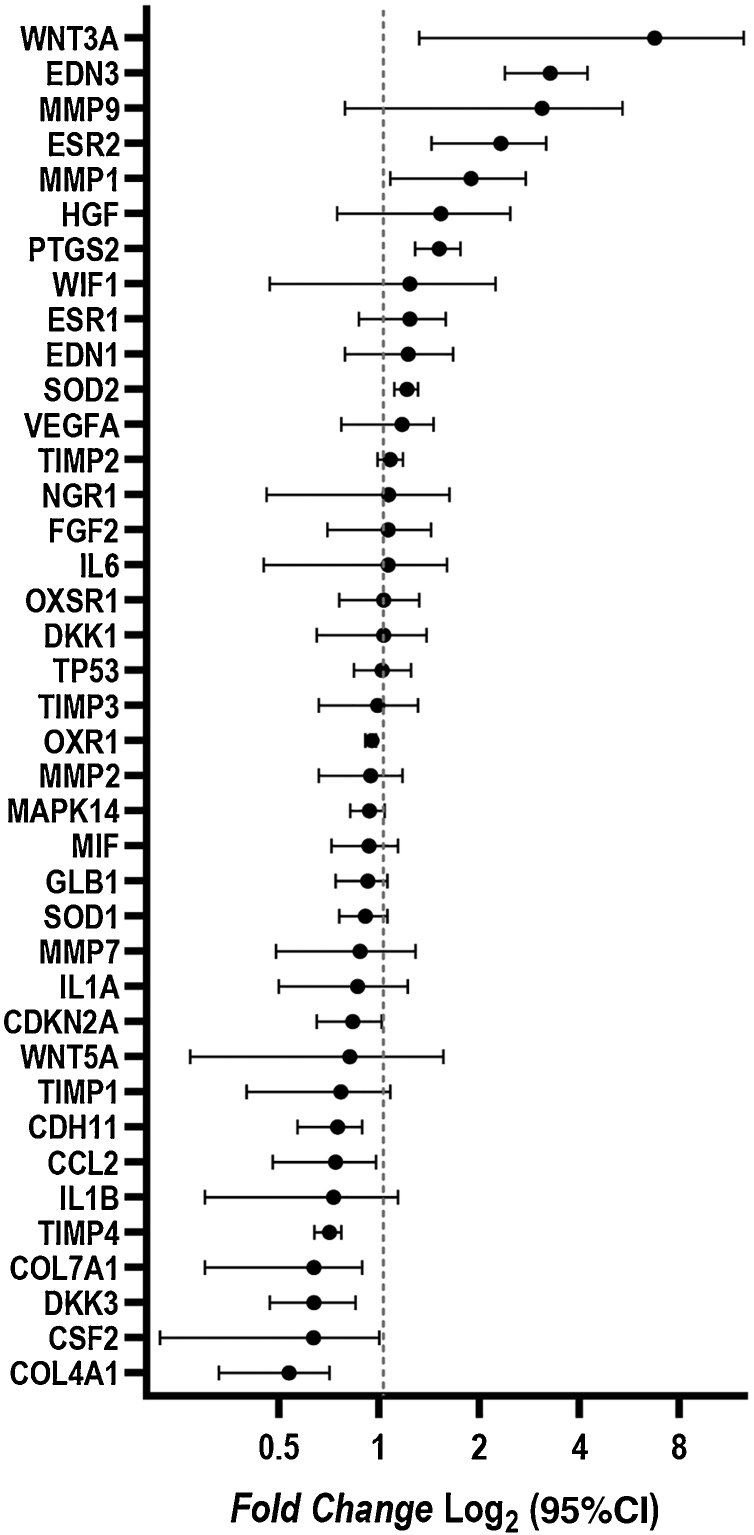
Fold change (mean Log_2_ and 95% CI) of the gene expression between skin with melasma and adjacent healthy skin assessed by real-time PCR array (n = 5).

Changes in the gene expression were identified in fibroblasts isolated from melasma skin when compared to those isolated from adjacent healthy skin. The *WNT3A, EDN3, ESR2, PTG2, MMP1,* and *SOD2* genes were up-regulated; *COL4A1, CSF2, DKK3, COL7A1, TIMP4, CCL2,* and *CDH11* genes were down-regulated (Fig. 4).

## Discussion

Melanogenesis is a complex process mediated by paracrine, autocrine, and environmental stimuli.^13^ The phenotypic changes seen in melasma skin are not primarily or solely due to epidermal alterations, as several dermal changes are evident in the affected skin when compared to adjacent healthy, photoexposed skin.^14^

Fibroblasts are the most common cell type in the dermis and, due to their great longevity, they accumulate damage to their cell machinery, which can result in functional and morphological alterations.^10^ Intrinsic aging and photoaging are associated with a decrease in fibroblast number and rate of proliferation; in this context, the comparison of the gene profile of fibroblasts originating from the skin with melasma with that of healthy adjacent photoexposed area reinforces that the gene alterations are independent of age and photoexposure.^3^, ^15^

Considering the importance of dermal fibroblasts in pigmentation regulation, the modifications of their autocrine and paracrine activities can influence pigmentation disorders.^10^ In the present study, the loss of fusiform morphology, higher amount of cytoplasmic SA-β-gal, and lower growth rate of fibroblasts from the skin with melasma were identified. These morphological and metabolic changes support a senescent phenotype in melasma skin fibroblasts.

Senescence is a central factor in the mechanisms of aging, and the Senescence-Associated Secretory Phenotype (SASP) is known to be the main trigger of age-related phenotypes, such as wrinkles and pigmentation.^16^ It should be noted that there is evidence of phenotypic differences related to ethnicity and susceptibility to fibroblast senescence.^17^

Senescent fibroblasts secrete inflammatory cytokines and stem cell factors, which promote collagen degradation through MMP activation and have reduced mitotic potential. There is increased stromal degradation and impaired tissue repair, as evidenced in the dermis of melasma. Therefore, a mosaic structure of tissue susceptibility to senescence can be theorized for the development of melasma in the skin under environmental stimuli such as UV radiation and sex hormones.^18^

There was an increase in MMP1 gene expression and a decrease in collagen IV, VII (*COL4A1, COL7A1*), and *TIMP4* expression in fibroblasts isolated from melasma skin. During the senescence process, there is a progressive increase in metalloproteinase production and a decrease in the repair process.^10^ Moreover, in dermal fibroblasts, α-MSH promotes the upregulation of interstitial collagenase and attenuates TGFβ1-induced collagen synthesis. Damage to the upper dermis and the basement membrane zone facilitates the transit of melanogenic factors to the basement layer. In the present study, despite a marginal implication, *MMP9* showed a high fold change (>2), which, in association with the lower expression of *TIMP1* and *4*, suggests participation of melasma skin fibroblasts in the deficient repair of the upper dermis and angiogenesis.

The role of sex hormones in cutaneous estrogen receptor β expression in melasma is well established.^19^ Overall, *ESR2* is expressed during the repair process.^20^ The upper dermis and basement membrane are heavily damaged in melasma skin, compared to adjacent healthy, photoexposed skin and photoprotected skin.^1^, ^14^ Moreover, estrogen, α-MSH, and HGF stimulate melanogenesis by directly binding to the melanocyte receptor and are also released during the wound healing process.^21^, ^22^ These findings indicate that the chromic and unsuccessful upper dermis repair process can induce melanogenesis in melasma.

Some genes related to the Wnt/β-catenin pathway showed different behavior in fibroblasts from the skin with melasma, in relation to photoexposed adjacent skin; the DKK3 inhibitory factor was down-regulated, while WNT3A was overexpressed. WNT3A plays an important role in controlling the proliferation and differentiation of melanocyte precursors and angiogenesis. In mature melanocytes, WNT3A increases the amount of melanin and tyrosinase activity.^23^ The Wnt pathway is involved in the pathophysiology of melasma, and the decreased expression of the inhibitory factor DKK3 in fibroblasts may influence the epidermal activation of WNT1.^4^ Moreover, WNT3A is important for the tissue repair process and can be activated due to damage to the upper dermis.^24^

EDN3 is a 21-amino acid peptide and preferentially activates EDNRB.^25^ EDN3 signaling is important in the normal development of epidermal and choroidal melanocytes, acting during cell proliferation, differentiation, and survival.^26^ Keratinocytes exposed to ultraviolet B radiation increase EDN production and secretion, causing adjacent melanocytes to activate the melanogenic cascade. Additionally, melanocytes exposed to this same radiation have an increased expression of c-KIT and EDNRB.^26^ EDN3 also participates in fibroblast chemotaxis, independently of prolonging cell survival and differentiation.^27^

Senescent fibroblasts are associated with impaired immune responses and skin tumor suppression, but SASP can vary among tissues and stimulus types.^28^ CSF participates in melanocyte growth, differentiation and survival, as well as in stem cell recruitment. Keratinocytes exposed to type A UVR *in vitro* have a higher level of CSF.^29^ Moreover, IL-1 produced by keratinocytes induces fibroblasts to synthesize CSF, which promotes keratinocyte proliferation and differentiation. A decrease in CSF in senescent fibroblasts can configure a reduction in keratinocyte stimuli, which hinders damaged tissue repair.^30^

CCL2 is a chemokine that acts as a critical regulator of macrophage and stem cell recruitment during wound healing, cancer, and infections.^31^ After cell stimulation with lipopolysaccharide, there is a constitutional down-regulation of *CCL2* in melanocytes from melanodermic individuals in comparison with melanocytes from individuals with a low phototype.^32^ The *CCL2* expression profile in fibroblasts can induce the darker focal phenotype of melasma.

Proteins from the cadherin family play an important role in intercellular adhesion and extracellular matrix synthesis regulation, as well as contributing to the signaling of events that control cell homeostasis.^33^ In skin fibroblasts, CDH11 regulates collagen and elastin synthesis, and its down-regulation can influence a decrease in dermal repair in melasma skin.^34^

Superoxide dismutase (SOD) is part of the enzymatic antioxidant system and is the main enzyme responsible for scavenging oxygen free radicals through the conversion of superoxide anions into hydrogen peroxide and oxygen.^35^ SOD is a complex of intracellular enzymes, and the serum level of SOD is higher in patients with melasma when compared to controls, which indicates an increase in the systemic oxidative status and reinforces the use of antioxidants in the treatment of melasma.^36^ SOD2 is a tetrameric mitochondrial enzyme that contains manganese and is targeted to the mitochondrial intermembrane space. The increase in SOD2 identified in fibroblasts is induced by an oxidative stress environment and may be the result of multiple inflammatory cytokines; moreover, it is a characteristic phenotype in senescent fibroblasts.^35^, ^37^

The fibroblast is the skin cell that contains the most PTGS. This enzyme participates in the synthesis of prostaglandin E2 (PGE2) from arachidonic acid, and PGE2 inhibits collagen production by fibroblasts *in vitro*.^38^ PTGS induction is significant in senescent cells, which increases the release of PGE2, leading to a reduction in type I collagen synthesis, vasodilation, and chemotaxis.^38^ In addition, PGE2 can directly induce melanogenesis and increase dendrites in melanocytes after UV irradiation.^39^, ^40^ The pro-inflammatory phenotype of fibroblasts in melasma can induce damage to the upper dermis and sustained melanogenesis.

This study has limitations related to the small sample size and the lack of proteomic or functional assays to confirm the findings. However, the results were consistent and allowed the authors to explore, in a preliminary way, the hypothesis about the role of fibroblasts in the pathogenesis of melasma, proposing new investigations related to the pathogenesis and therapeutic strategies of melasma. The sample of patients submitted to the senescence and proliferation assay was different from the one submitted to the gene expression comparison, although the results were parallel. Finally, the investigation of fibroblasts from controls without melasma, matched by age, photoexposure pattern, photoaging, and phototype, could also elucidate the particular susceptibility of fibroblasts from individuals with melasma to senescence.

Further studies should investigate the regulatory factors of these pathways and the cytokine network between keratinocytes, melanocytes, nerves, endothelium, and fibroblasts in melasma, preferably through studies with tissue cultures, for genomic, proteomic, and functional experiments.

## Conclusions

Fibroblasts from the skin with melasma showed a lower growth rate, less fusiform morphology, and greater accumulation of SA-β-gal than those from adjacent healthy photoexposed skin. Furthermore, their gene expression profile comprised pro-inflammatory, pro-melanogenic, and tissue repair deficit-related factors, which can induce damage to the upper dermis and support the focal pigmentary phenotype in melasma.

## Financial support

FAPESP – number 2012/09233-5, 2012/05004-1; CNPq – number 401309/2016-9.

## Authors' contributions

Ana Cláudia Cavalcante Espósito: Design and planning of the study; collection, analysis, and interpretation of data; drafting and editing of the manuscript; Critical review of the literature; Approval of the final version of the manuscript.

Gabrielli Brianezi: Design and planning of the study; collection, analysis, and interpretation of data; approval of the final version of the manuscript.

Luciane Donida Bartoli Miot: Design and planning of the study; collection, analysis, and interpretation of data; effective participation in research orientation; approval of the final version of the manuscript.

Hélio Amante Miot: Design and planning of the study; collection, analysis, and interpretation of data; statistical analysis; drafting and editing of the manuscript; critical review of the literature; critical review of the manuscript; approval of the final version of the manuscript.

## Conflicts of interest

None declared.
